# Systemic AAV9 Gene Therapy Mitigates Neuromuscular Junction Degeneration and Muscle Atrophy in a Mouse Model of CLN1 Disease

**DOI:** 10.3390/ijms27073080

**Published:** 2026-03-28

**Authors:** Ewa A. Ziółkowska, Albina Jablonka-Shariff, Letitia L. Williams, Elizabeth M. Eultgen, Matthew D. Wood, Daniel A. Hunter, Mark S. Sands, Alison K. Snyder-Warwick, Jonathan D. Cooper

**Affiliations:** 1 Department of Pediatrics, Washington University School of Medicine, St. Louis, MO 63110, USA; ziolkowska.phd@gmail.com (E.A.Z.); l.l.williams@wustl.edu (L.L.W.); e.eultgen@wustl.edu (E.M.E.); 2 Department of Surgery, Washington University School of Medicine, St. Louis, MO 63110, USA; jablonka@med.umich.edu (A.J.-S.); woodmd@wustl.edu (M.D.W.); hunterd@wustl.edu (D.A.H.); snyderwa@med.umich.edu (A.K.S.-W.); 3 Department of Medicine, Washington University School of Medicine, St. Louis, MO 63110, USA; mssands@wustl.edu; 4 Department of Genetics, Washington University School of Medicine, St. Louis, MO 63110, USA; 5 Department of Neurology, Washington University School of Medicine, St. Louis, MO 63110, USA

**Keywords:** CLN1 disease, neuromuscular junction, peripheral nervous system, muscle atrophy, terminal Schwann cells, AAV9 gene therapy

## Abstract

CLN1 disease, caused by mutations in the PPT1 gene, is a fatal neurodegenerative lysosomal storage disorder. While central nervous system (CNS) pathology is well documented, the impact on peripheral tissues remains unclear. Having previously described severe spinal cord pathology, we investigated whether PPT1 deficiency also impacts the neuromuscular junction (NMJ) and skeletal muscle, and whether early systemic gene therapy can prevent these disease manifestations. NMJ morphology, terminal Schwann cell (tSC) coverage, and skeletal muscle structure were examined in symptomatic and end-stage *Ppt1^−/−^* mice. Neonatal mice received systemic AAV9-hCLN1 gene therapy via intravenous injection. Untreated *Ppt1^−/−^* mice exhibited pronounced NMJ pathology, including progressive tSC loss, apparently reduced innervation, and increased abnormal acetylcholine receptor clustering. In parallel, we observed skeletal muscle atrophy, with decreased myofiber diameter and reduced myonuclear content, despite preserved sciatic nerve morphology. Systemic AAV9-hCLN1 therapy partially prevented or ameliorated these phenotypes, preserving NMJ innervation and muscle fiber structure. These findings identify peripheral NMJ and muscle abnormalities as previously unrecognized features of CLN1 disease and provide proof-of-concept that early systemic gene therapy can mitigate these effects. Our results highlight the systemic nature of CLN1 pathology and support the need for treatments that address both CNS and peripheral targets for comprehensive disease modification.

## 1. Introduction

CLN1 disease, also known as infantile neuronal ceroid lipofuscinosis (INCL), is a severe and rapidly progressive lysosomal storage disorder [1,2,3,4], which belongs to the broader group of neurodegenerative conditions known as the neuronal ceroid lipofuscinoses (NCLs), or Batten disease [5,6,7,8]. CLN1 disease is caused by mutations in the *CLN1/PPT1* gene, which encodes palmitoyl-protein thioesterase 1 (PPT1) [9], a lysosomal enzyme responsible for removing long-chain fatty acids from lipid-modified proteins [10]. Via mechanisms that remain poorly understood, loss of PPT1 function leads to the accumulation of autofluorescent storage material within lysosomes, especially in neurons, ultimately resulting in widespread neurodegeneration, motor and cognitive decline, seizures, and early death [1,4,11,12,13].

Based on this neurological presentation, CLN1 disease has been considered primarily as a central nervous system (CNS) disorder [1,4,8]. Indeed, most studies to date have focused on defining the effects of PPT1 deficiency upon the brain and spinal cord [14,15,16,17,18,19,20], where early activation of glial cells and progressive neuronal loss are prominent features. We recently defined that spinal cord pathology occurs very early in disease progression [18,19], including changes in innervation in the dorsal horn and a significant loss of ventral horn motor neurons along the entire length of the spinal cord in PPT1-deficient mice (*Ppt1^−/−^* mice). These findings prompted us to investigate the extent of pathological changes within the peripheral nervous system (PNS), focusing on the sciatic nerve, the innervation of the neuromuscular junction (NMJ), and skeletal muscle in the lower limb. We also characterized terminal Schwann cells (tSCs), a specialized type of glial cell located at the NMJ, which play key roles in maintaining synaptic stability, supporting axon terminals, and guiding reinnervation after injury [21,22]. All these structures, including tSCs, have recently been reported to be affected pathologically in a mouse model of CLN3 disease [23], another form of NCL, but are yet to be investigated in CLN1 disease.

The monogenic nature of CLN1 makes it a suitable candidate for gene therapy [24], with transduced cells secreting PPT1 enzyme that can be taken up by neighboring deficient cells via a receptor-dependent uptake mechanism [25,26,27]. Several preclinical studies have demonstrated that CNS-directed adeno-associated virus serotype 9 (AAV9)-mediated delivery of *PPT1* can prevent many of the neuropathological and behavioral phenotypes of murine CLN1 disease [17,28,29,30,31,32,33], slow disease progression, and extend lifespan, especially when administered to both the brain and spinal cord [18]. More recently, intravenous AAV9 delivery has shown the capacity to transduce peripheral tissues and prevent pathology in extra-CNS compartments such as the enteric nervous system (ENS) [34]. However, whether such systemic gene therapy can address neuromuscular dysfunction or skeletal muscle pathology in CLN1 disease has not yet been investigated.

In this study, we examined the effects of CLN1 deficiency on the sciatic nerve, NMJ, and skeletal muscle using *Ppt1^−/−^* mice at symptomatic and end-stage timepoints. Unexpectedly, very little evidence for sciatic nerve axon loss or demyelination was found even at disease endstage. However, we found profound neuromuscular abnormalities in untreated *Ppt1^−/−^* mice, including progressive tSC loss, apparent NMJ denervation, and muscle fiber atrophy. We then tested whether systemic gene therapy with an AAV9 virus expressing human CLN1 (AAV9-hCLN1), delivered during the early postnatal period, could prevent or ameliorate these deficits. Our results show that gene therapy transduced a subset of myofibrils and preserved NMJ structure and improved muscle integrity in AAV9-hCLN1-treated *Ppt1^−/−^* mice. These findings reveal previously underrecognized neuromuscular manifestations of CLN1 disease and highlight the importance of addressing both central and peripheral targets in therapeutic strategies.

## 2. Results

### 2.1. No Evidence of Sciatic Nerve Axonal Loss or Demyelination

**Figure 1:**
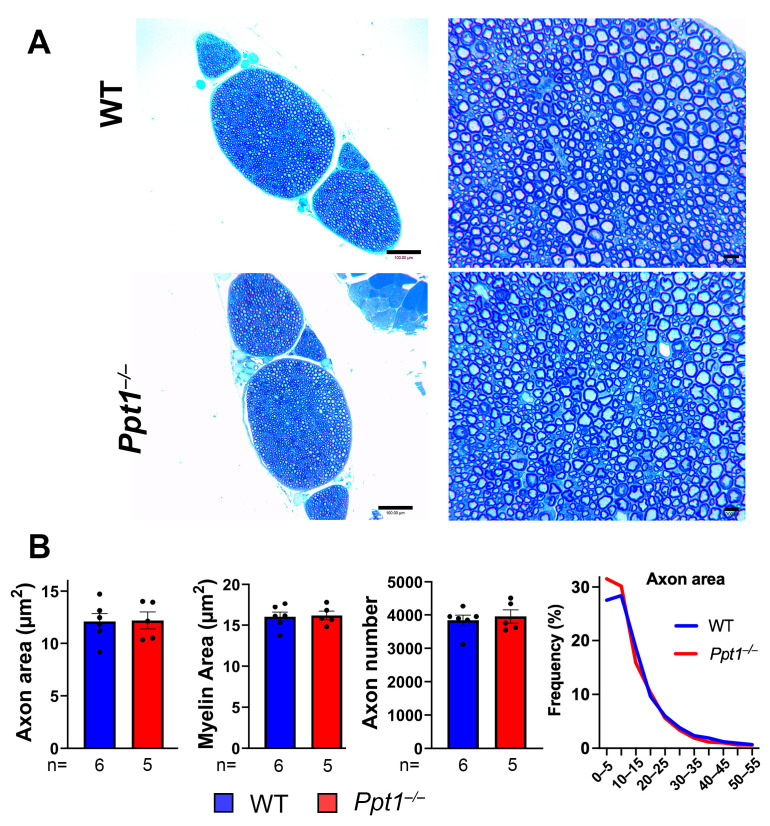
Sciatic nerve morphology and myelination are preserved in *Ppt1^−/−^* mice at disease endstage. (**A**) Representative toluidine blue-stained cross-sections of sciatic nerves from untreated wild-type (WT) and *Ppt1^−/−^* mice at 7 months of age, representing disease endstage. Both low- and high-magnification images show no overt differences in overall nerve organization, axonal density, or myelin structure between genotypes. Scale bar = 100 µm for lower power view; 10 µm for higher power view. (**B**) Quantification of average axon area (and its distribution), myelin area, and axon number, in sciatic nerves from WT and *Ppt1^−/−^* mice. No significant differences were observed across groups for any of the measured parameters at disease endstage. Data are presented as mean ± SEM. Each dot represents an individual mouse. Unpaired *t*-test revealed no significant differences between genotypes. Representative images are shown; quantitative analyses were performed on all analyzed nerves, with *n* values indicated below the *x*-axis.

Both motor and sensory components of the spinal cord degenerate progressively in *Ppt1^−/−^* mice [18,19]. To investigate whether peripheral nerves are also structurally affected by PPT1 deficiency, we performed morphological and quantitative analyses of sciatic nerve cross-sections from WT and *Ppt1^−/−^* mice at 7 months of age. No gross anatomical differences in overall nerve organization or axonal density between WT and *Ppt1^−/−^* samples were observed (Figure 1A). Mice of both genotypes exhibited comparable nerve bundle architecture, axonal packing, and myelin sheath appearance. Quantitative assessment of axonal parameters, including total axon number, individual axon area and its size distribution, and myelin area, further confirmed the absence of any detectable structural deficits (Figure 1B). No statistically significant differences were observed for any parameter across experimental groups. Average axon diameter and myelin thickness remained stable in *Ppt1^−/−^* mice, indicating intact myelination and axonal preservation in the PNS even at disease endstage. These findings suggest that PPT1 deficiency does not result in overt degeneration or demyelination of the sciatic nerve, although ultrastructural analyses would be needed to substantiate this. Similar results were obtained from *Thy1-YFP/Ppt1^−/−^* mice analyzed in parallel, supporting the conclusion that peripheral axon structure is largely preserved even at the terminal stage of disease progression in this CLN1 mouse model.

### 2.2. Progressive tSC Loss and NMJ Denervation Is Partially Preserved by Systemic AAV9-hCLN1 Gene Therapy in Ppt1^−/−^ Mice

**Figure 2:**
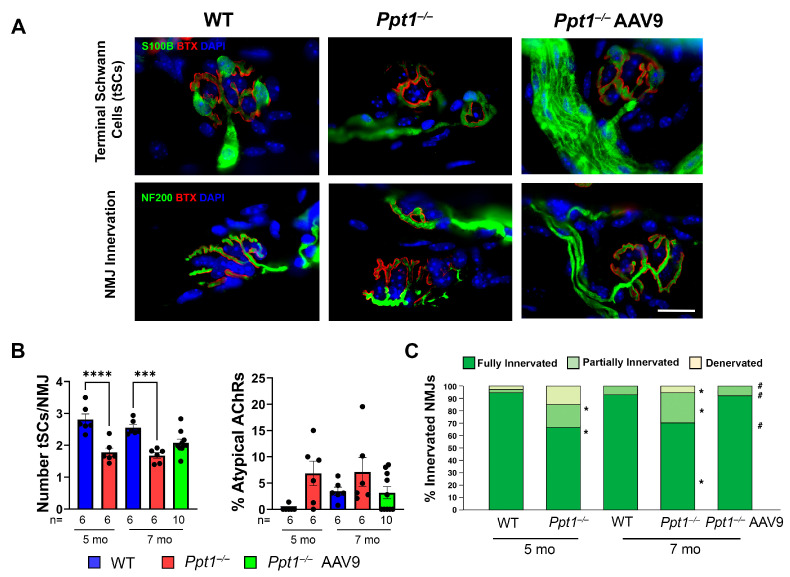
Systemic AAV9-hCLN1 gene therapy preserves NMJ structure and terminal Schwann cell number in *Ppt1^−/−^* mice. (**A**) Representative confocal images of neuromuscular junctions from EDL muscle in wild type (WT), untreated *Ppt1^−/−^*, and AAV9-hCLN1-treated *Ppt1^−/−^* mice at 7 months (mo) of age. NMJs were labeled with antibodies against terminal Schwann cells (S100B, green), axons (NF200, green), and acetylcholine receptors (AChRs stained with α-BTX, red), with DAPI (blue) marking nuclei. Untreated *Ppt1^−/−^* mice show reduced tSC coverage, disrupted AChRs clustering, and partial NMJ denervation, while treated mice exhibit improved structural preservation of NMJ components. (**B**) Quantification shows a significant decrease in the number of tSCs per NMJ in untreated *Ppt1^−/−^* mice at 5 and 7 months, with partial amelioration following AAV9-hCLN1 treatment. The percentage of NMJs with abnormal AChR morphology was increased in untreated mice and slightly reduced following gene therapy. (**C**) Innervation analysis demonstrates a marked decline in fully innervated NMJs and a rise in partially innervated and denervated junctions in untreated *Ppt1^−/−^* mice, both of which were partially corrected by systemic gene therapy. NMJs were considered fully innervated when ≥75% of the endplate was occupied by axonal staining. Data are shown as mean ± SEM. Each dot represents one mouse. One-way ANOVA with Bonferroni post hoc test. * *p* ≤ 0.05, *** *p* ≤ 0.001, **** *p* ≤ 0.0001 (vs. WT); ^#^ *p* ≤ 0.05 (vs. untreated *Ppt1^−/−^*). Scale bar: 20 μm (**A**). Representative images are shown, with the number of mice (*n*=) per treatment group indicated below the *x*-axis.

Motor nerves innervate the NMJ, where terminal Schwann cells (tSCs) regulate synaptic stability [21,22,35]. To determine if these structures are pathologically affected in murine CLN1 disease, we analyzed tSCs and NMJ innervation in the EDL muscle of WT, untreated *Ppt1^−/−^*, and AAV9-hCLN1-treated *Ppt1^−/−^* mice at 5 and 7 months of age, representing symptomatic and disease endstage, respectively. Confocal imaging of EDL muscle revealed a marked reduction in the number of tSCs per NMJ in *Ppt1^−/−^* mice compared to WT controls at both ages (Figure 2A, top panels). Quantification confirmed a significant decrease in tSC number in *Ppt1^−/−^* mice at 5 and 7 months (Figure 2B, left) and an increase in fragmented tSCs, suggesting potential disruption of glial support at the synapse. This was accompanied by an increase in morphologically abnormal endplates in *Ppt1^−/−^* mice at both ages, using α-BTX staining of AChR clustering (Figure 2B, right).

We next assessed the degree of NMJ innervation using dual labeling of axons (NF200) and α-BTX. In WT mice, most NMJs were fully innervated at both 5 and 7 months (Figure 2A, bottom left). In contrast, *Ppt1^−/−^* mice displayed clear signs of apparent innervation defects, with fragmented axonal terminals and incomplete overlap with α-BTX (Figure 2A, bottom right). Quantitative analysis revealed a significant reduction in the proportion of fully innervated NMJs and a corresponding increase in partially innervated and denervated junctions in *Ppt1^−/−^* mice at both time points (Figure 2C). These results demonstrate progressive NMJ degeneration in the absence of PPT1, coinciding with tSC loss and postsynaptic NMJ disorganization during the disease course.

To assess the therapeutic efficacy of neonatal intravenous AAV9-hCLN1 gene therapy on peripheral synaptic integrity, we examined NMJ morphology and tSCs in EDL muscles in AAV9-hCLN1-treated *Ppt1^−/−^* mice at disease endstage. This vector is essentially that used in our neuromuscular studies in CLN3 mice, except that it expresses PPT1 instead of either CLN3 or GFP, which transduces a proportion of tSCs and robustly transduces skeletal muscle [23]. Confocal imaging showed that AAV9-treated *Ppt1^−/−^* mice retained higher numbers of tSCs per NMJ compared to untreated *Ppt1^−/−^* mice (Figure 2A, top), with many NMJs exhibiting near-normal tSC coverage and structure. Quantification confirmed the partial preservation of tSC numbers in AAV9-hCLN1-treated *Ppt1^−/−^* mice, with values significantly higher than those of untreated *Ppt1^−/−^* mice, although still below WT levels (Figure 2B, left).

Despite improvements in tSC coverage, the percentage of abnormal AChR clusters remained modestly elevated in both AAV9-hCLN1-treated and untreated *Ppt1^−/−^* mice at 7 months of age, suggesting that postsynaptic defects are only partially ameliorated by gene therapy (Figure 2B, right). Nevertheless, the apparent NMJ denervation in *Ppt1^−/−^* mice was significantly reduced by AAV9-hCLN1-treatment of *Ppt1^−/−^* mice. Immunostaining for NF200 and α-BTX revealed a substantial increase in the proportion of fully innervated NMJs in AAV9-hCLN1-treated *Ppt1^−/−^* mice compared to untreated *Ppt1^−/−^* littermates (Figure 2A, bottom). Quantitative analysis confirmed this observation, showing a significant shift toward full innervation and corresponding reductions in the proportion of partially innervated and denervated NMJs (Figure 2C). These results indicate that systemic AAV9-mediated CLN1 gene replacement therapy can preserve NMJ architecture and prevent synaptic disconnection at the terminal stage of disease.

### 2.3. Ppt1 Deficiency Leads to Muscle Fiber Atrophy and Reduced Myonuclear Content in Skeletal Muscle, with Partial Amelioration by Systemic AAV9-hCLN1 Gene Therapy

**Figure 3:**
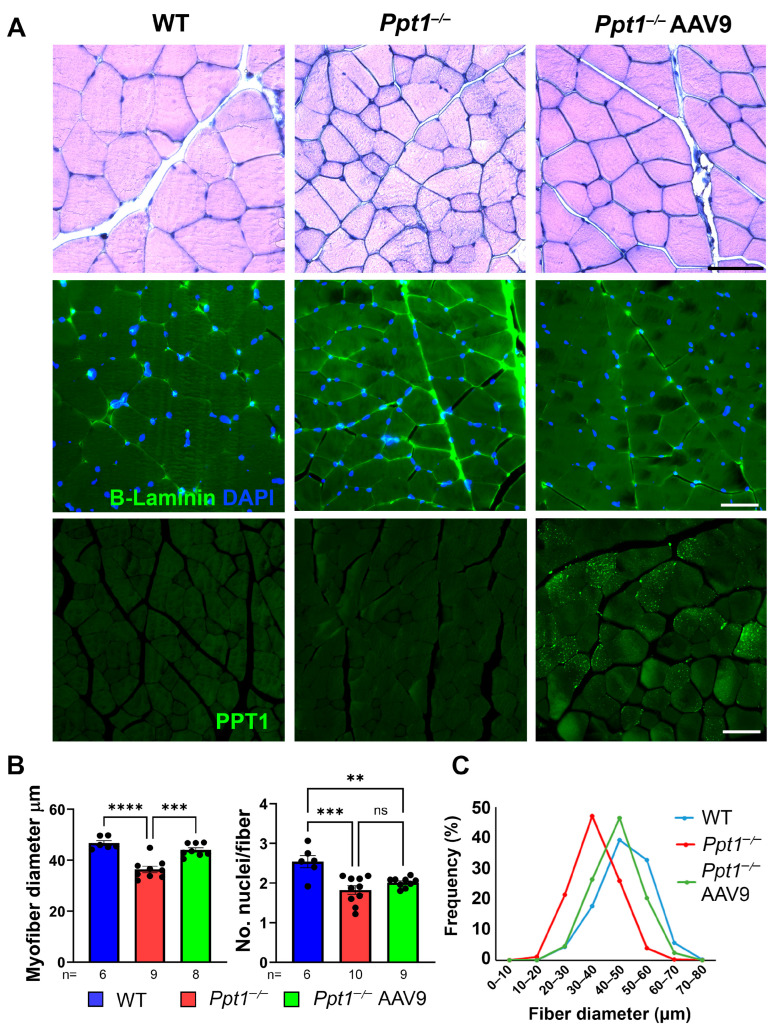
AAV9-hCLN1 gene therapy improves skeletal muscle architecture in *Ppt1^−/−^* mice (**A**) Representative images of quadriceps muscle cross-sections from wild type (WT), untreated *Ppt1^−/−^*, and AAV9-treated *Ppt1^−/−^* mice at 7 months of age. Immunostaining for laminin (green, middle row) and DAPI (blue, middle row) reveals significantly reduced fiber diameter and disorganized fiber architecture in untreated *Ppt1^−/−^* muscle, with partial improvement following systemic AAV9-hCLN1 (*Ppt1^−/−^* AAV9) therapy. PPT1 immunostaining reveals the transduction of a subset of myofibrils in AAV9-hCLN1-treated *Ppt1^−/−^* muscle, which displays bright punctate PPT1 immunoreactivity (green, bottom row), which is not present in untreated *Ppt1^−/−^* muscle. (**B**) Quantification reveals a significant reduction in the average number of nuclei per myofiber and decreased myofiber diameter in untreated *Ppt1^−/−^* mice compared to WT controls. AAV9 treatment partly prevented decreased myofiber diameter and showed a trend toward increased myonuclear content. (**C**) Fiber size distribution curve indicates a shift to smaller size in untreated *Ppt1^−/−^* mice, with a partial preservation of myofiber diameter in AAV9-treated *Ppt1^−/−^* mice. Data are presented as mean ± SEM. Each dot represents one mouse. One-way ANOVA with Bonferroni post hoc correction; ** *p* ≤ 0.01, *** *p* ≤ 0.001, **** *p* ≤ 0.0001, ns = not significant. Scale bar = 50 μm (**A**). Representative images are shown; quantitative analyses were performed on ≥300 muscle fibers per mouse, with the number of mice (*n*=) per treatment group indicated below the *x*-axis.

To determine whether *Ppt1^−/−^* mice also exhibit peripheral muscular pathology at disease endstage, we analyzed cross-sections of quadriceps muscles from WT and *Ppt1^−/−^* mice at 7 months. Hematoxylin and eosin staining revealed a striking reduction in average myofiber size in *Ppt1^−/−^* mice compared to WT controls (Figure 3A, left panels). Higher-magnification images showed smaller myofibers and occasional angular atrophic fibers in *Ppt1^−/−^* muscle, while WT samples displayed uniformly large, polygonal fibers. Immunostaining for laminin and DAPI further confirmed this phenotype, highlighting shrunken fiber borders and reduced nuclear density in *Ppt1^−/−^* muscle (Figure 3A, right panels).

Quantitative analysis revealed a significant decrease in the average number of nuclei per muscle fiber in disease-endstage *Ppt1^−/−^* mice (Figure 3B, right), suggesting impaired myonuclear maintenance. In parallel, the average myofiber diameter was markedly reduced (Figure 3B, left), consistent with muscle atrophy. Fiber size distribution analysis confirmed a leftward shift to smaller myofiber diameter in *Ppt1^−/−^* mice (Figure 3C), with the majority of myofibers falling in the 30–40 μm diameter range, compared to the 40–50 μm diameter peak observed in WT controls (Figure 3C). These findings indicate that, in addition to central and neuromuscular pathology, PPT1 deficiency results in skeletal muscle atrophy and structural deterioration at disease endstage.

Given the muscle fiber atrophy observed in untreated *Ppt1^−/−^* mice (Figure 3), we next evaluated whether systemic AAV9-hCLN1 gene therapy could preserve skeletal muscle structure at the terminal disease stage. Immunostaining for laminin (green) and DAPI (blue) revealed clear histological improvement in quadriceps muscles from treated *Ppt1^−/−^* mice (Figure 3A). While untreated *Ppt1^−/−^* mice exhibited small, irregularly shaped muscle fibers, those from AAV9-hCLN1-treated mice showed a more uniform morphology resembling that of WT controls. These IV AAV9-hCLN1-treated *Ppt1^−/−^* mice also displayed intense punctate PPT1 immunoreactivity within a subset of myofibrils (Figure 3A), consistent with our previous data using an equivalent vector in CLN3 mice [23].

Quantification confirmed a significant restoration in both myofiber nuclear content and fiber diameter following AAV9-hCLN1 treatment (Figure 3B,C). The number of nuclei per myofiber in treated mice was significantly increased in AAV9-hCLN1-treated compared to untreated *Ppt1^−/−^* mice and did not differ from WT mice. Similarly, myofiber diameters were partially restored, though, on average, they remained slightly smaller than in WT mice. Myofiber size distribution analysis supported these findings, showing a rightward shift to higher myofiber diameter in AAV9-hCLN1-treated *Ppt1^−/−^* mice compared to untreated mutants (Figure 3C), with a peak myofiber diameter around 40–50 μm—more closely resembling the size distribution profile seen in WT mice (Figure 3B, right). These results demonstrate that AAV9-mediated CLN1 gene therapy not only preserves NMJ innervation (Figure 2) but also improves skeletal muscle structure and fiber size at disease endstage.

## 3. Discussion

CLN1 disease, caused by loss-of-function mutations in the *PPT1* gene [9], is a devastating neurodegenerative lysosomal storage disorder that has been defined by its profound CNS pathology [1,4,13]. How deficiency of this depalmitoylating enzyme results in such severe neuronal loss remains unclear, but a variety of different possible underlying mechanisms have been proposed [36,37,38]. These involve different inflammatory responses, unfolded protein responses, and impaired nutrient sensing, but none have been conclusively demonstrated as key to CLN1 pathogenesis [36,37,38]. Nevertheless, while progressive CNS pathology is undoubtedly central to disease pathogenesis and clinical decline, our findings reveal that CLN1 disease also affects components of the PNS and neuromuscular system. Despite very little evidence of peripheral nerve damage, our new data reveal pathological effects upon the NMJ and its innervation, the depletion of tSCs, which also appear morphologically compromised, and that these pathologies extend to skeletal muscle. Such pathologies may contribute significantly to disease burden and are unlikely to be treated by CNS-directed therapeutic approaches alone but appear to be treatable by systemic gene therapy. Furthermore, CNS-directed therapies may be insufficient to fully address the multisystemic nature of the disease in the rest of the body. CSF-directed delivery approaches, particularly using AAV9, have been shown to confer variable levels of peripheral transduction depending on dose, age at administration, and route of delivery. Indeed, based on our experience in treating both CNS and extra-CNS effects of disease in these disorders, it is likely that dual-delivery strategies designed to intentionally target both the brain and peripheral tissues will be required to achieve optimal therapeutic efficacy. These data reinforce the notion that the effects of CLN1 disease extend outside the CNS, and that targeting both central and peripheral pathologies is necessary for optimal treatment outcomes.

Given its overwhelming neurological presentation [1,4], the primary focus of mechanistic and therapeutic research in CLN1 disease has understandably centered on the brain and spinal cord [14,15,16,17,18,19,39], with treatments such as enzyme replacement and gene therapy being directed to the CNS [18,19,28,30,40,41]. We recently described the early onset of profound neurodegeneration in the spinal cord of PPT1-deficient mice [18,19], preceding the progressive microglial activation, astrocytosis, and widespread neuronal loss that occurs in the forebrain and cerebellum [14,15,16,17]. These neurodegenerative effects can be prevented to different extents by the ICV delivery of recombinant human PPT1 enzyme [42], or brain-directed AAV9-mediated gene therapy [18,28,29,30]. This latter approach is especially effective if both the brain and spinal cord are treated, with synergistic effects on behavior, pathology, and lifespan [18]. However, it has become increasingly apparent that life-limiting disease effects are also systemically apparent in multiple forms of NCL and are not ameliorated by such CNS-directed treatment strategies [23,34,43].

We recently reported progressive neurodegeneration in the enteric nervous system (ENS) of mouse models of CLN1 and CLN2 diseases, resulting in bowel dysmotility and contributing to premature death [34]. Profound ENS neuron loss is also evident in people with these types of NCL [34], and we have found similar phenotypes in human CLN3 disease and in the corresponding mouse model [44]. The effects of CLN3 deficiency also extend to the PNS [23], and this study was designed to determine if similar phenotypes exist in CLN1-disease mice. Based on our evidence of pronounced pathology in the dorsal and ventral spinal cord in *Ppt1^−/−^* mice [18,19], we anticipated that both sensory and motor components of the PNS would also be affected in these mice. This prompted us to analyze the sciatic nerve, which contains both sensory and motor axons, and we anticipated finding marked pathology in this nerve, secondary to these degenerative events in the spinal cord. Surprisingly, in semi-thin sections, we found very little evidence for either axon loss or overt demyelination in the sciatic nerve and instead uncovered evidence for pathological effects at NMJ nerve terminals. It will be important to conduct ultrastructural analyses of these nerves, alongside teased fiber preparations and node/paranode immunostaining, to determine if more subtle morphological abnormalities are apparent. Nevertheless, the relative preservation of sciatic nerve morphology in the presence of pronounced neuromuscular junction and muscle pathology suggests differential vulnerability within the motor system. Distal synaptic sites, such as the neuromuscular junction, may be affected prior to overt degeneration of the peripheral nerve, consistent with a synaptopathic or dying-back process. To substantiate these observations and shed more light on possible mechanisms, it will be important to assess synaptic vesicle markers, such as SV2 or synaptophysin, or active zone proteins, such as bassoon, in our mice. While our data on sensory nerve endings will be reported separately, in this study, we report novel evidence for profound effects of PPT1 deficiency at NMJs of the lower limb. As our data demonstrate, these newly discovered NMJ phenotypes include apparently reduced NMJ innervation, which itself shows morphological abnormalities, and the loss and morphological disruption of tSCs that are key for the initial formation and maintenance of NMJ stability [21,22,35]. These data resemble those we have recently reported in CLN3-deficient mice [23], suggesting NMJ structures are affected consistently across multiple forms of NCL.

A key issue that remains unresolved is the underlying mechanistic basis of these effects upon the neuromuscular system. The primary enzymatic deficiency in CLN1 disease may potentially impair cell-autonomous functions in muscle fibers and glial cells, particularly given the established role of PPT1 in regulating protein palmitoylation and lipid homeostasis, processes that influence membrane stability and susceptibility to oxidative damage [37]. Alternatively, NMJ disruption may arise secondarily to CNS motor neuron pathology or systemic inflammation. We plan to explore both of these possibilities mechanistically to address questions regarding the cellular autonomy of PPT1 deficiency and to explore whether muscle pathology represents primary myopathy or occurs secondary to CNS pathology.

Our findings of both glial cells and skeletal muscle involvement, in the context of preserved axonal integrity, point toward multiple peripheral cell types being vulnerable to PPT1 deficiency. Retraction of nerve terminals at the NMJ may potentially occur as a result of dysfunctional or dying spinal motor neurons [18,19], although the loss of tSCs may also be a key influence, given the normal function of these cell types at the NMJ [21,22,35]. Indeed, since tSCs are critical for NMJ maintenance, axon guidance, and reinnervation following injury, their depletion may represent a primary driver of NMJ instability and denervation [21,22,35]. This loss of tSCs, as the main cell type present at NMJs, is consistent with the effects of PPT1 deficiency upon CNS glial cells [45], which reveal both astrocytes and microglia to be dysfunctional in *Ppt1^−/−^* mice. As such, our data raises the possibility that glial cells outside the CNS are also affected by PPT1 deficiency, and it will be important to determine whether tSCs are functionally compromised in *Ppt1^−/−^* mice.

Both tSC loss and NMJ innervation defects were already evident in symptomatic *Ppt1^−/−^* mice at 5 months of age and did not substantially worsen at disease endstage, although a greater proportion of NMJs showed morphological abnormalities at this 7-month time point. Establishing cause and effect between these events and studying cellular autonomy of PPT1 deficiency at the NMJ is complicated by the normal release of PPT1 and its uptake by mannose-6-phosphate receptors on neighboring deficient cells [25,26,27]. It is likely that we will need to employ a membrane-tethered approach to facilitate the generation of cell-type-specific PPT1 deficiency, as we have recently employed for the TPP1 enzyme deficient in CLN2 disease [46].

Another possibility that our data reveals is a direct effect of PPT1 deficiency on skeletal muscle, consistent with our data from CLN3 disease [23]. This includes myofiber atrophy and an apparent reduction in myofiber nuclei, although we cannot exclude that this may include some loss of satellite or interstitial cell nuclei, which are not discriminated from myonuclei by DAPI staining. Although weakness, spasticity, and muscle wastage are underlying features of CLN1 disease [1,4], the underlying causes of these phenotypes remain unknown. Importantly, sciatic nerve morphology and myelination appeared grossly intact in our analyses, suggesting that muscle pathology is not simply a downstream consequence of peripheral axon loss, but may involve events at the NMJ or direct effects of disease upon myofibers. Children with all forms of NCL display muscular atrophy at the latter stages of disease progression, but muscle phenotypes do not feature prominently in the earlier stages of disease [1,4]. Indeed, such effects on the muscle have been considered secondary to poor mobility or nutrition, or potentially being neurogenic in nature, as a result of decreased input from the CNS. Our data for tSC loss may reveal a cellular basis for NMJ denervation, with decreased nerve firing and muscle use, plausibly leading to its atrophy. However, functional measurements of NMJ and muscle potentials in *Ppt1^−/−^* mice are currently lacking and will be required to resolve whether this is the case and whether functional defects can also be rescued by gene therapy, and such experiments are now underway.

An alternative explanation of our data is that the muscle atrophy we have documented could contribute to degenerative events at the NMJ. This theory is consistent with events that occur in primary myopathies in which post-synaptic disease effects upon skeletal muscle result in NMJ phenotypes that resemble our data [47,48]. In other lysosomal disorders, including Pompe disease and metachromatic leukodystrophy (MLD), primary or secondary myopathy has been implicated in disease progression [49,50]. We recently reported pronounced evidence of myopathy in an early-stage muscle biopsy from a child with CLN3 disease [23]. Such effects need to be substantiated across more cases, and in other forms of NCL, including CLN1 disease. However, such biopsies are not routinely collected from children with any form of NCL, and mechanistic studies in mice are needed to determine whether the muscle phenotype in CLN1 disease is neurogenic, myogenic, or both.

Regardless of the underlying cause, our data demonstrates that systemic neonatal AAV9-mediated gene therapy substantially prevents several of these peripheral pathologies. Even if these protective effects are not complete, treated *Ppt1^−/−^* mice displayed significantly improved NMJ innervation, increased numbers of tSCs, and restoration of myofiber diameter and nuclear content. Notably, postsynaptic AChR abnormalities at the NMJ were only modestly improved, suggesting that early treatment may preserve NMJ connectivity but not fully normalize its architecture once these defects are established. Nonetheless, these findings provide strong proof-of-concept that neuromuscular phenotypes in CLN1 disease are modifiable with systemic gene therapy. An important consideration is how these treatment effects are mediated, but this is hard to determine conclusively in the case of AAV9-mediated delivery of PPT1. This is because the lysosomal enzyme is secreted from transduced cells and taken up by nearby cells via receptor-mediated uptake, a process termed ‘cross-correction’ [26]. We have previously reported that the AAV9 viral vector used in this study transduces a subset of tSCs, but also highly transduces skeletal muscle [23]. This distribution of cellular transduction may explain the therapeutic effects upon the NMJ of delivering the CLN3 transmembrane protein [23], which is not secreted or taken up like PPT1. In this study, we substantiated these findings, revealing the robust presence of PPT1 immunoreactivity in a subset of myofibrils, which may be released and taken up by neighboring myofibrils and tSCs to cross-correct their PPT1 deficiency. In the absence of PPT1 enzyme activity assays, it is not clear why this gene therapy strategy does not rescue the apparent reduction in myonuclear number. However, transducing a higher proportion of myofibers and/or higher levels of PPT1 expression may be required to achieve this. We are also exploring a range of approaches to improve the efficacy of treating the neuromuscular system. These may include, but are not limited to, muscle-specific promoters, different capsid variants, and expressing modified forms of PPT1 that have favorable binding and uptake properties.

Another key consideration is whether the treatment effects we have documented in the neuromuscular system occur secondary to treating the CNS, even partly. Our recent data suggest that AAV9-hPPT1 delivered neonatally to the superior temporal vein (as in this study) results in remarkably little transduction of the CNS of *Ppt1^−/−^* mice [34], which is mostly confined to the cerebral cortex. This treatment results in relatively low levels of PPT1 enzyme activity compared to direct AAV9-hPPT1 delivery to the CNS [18], and correspondingly, only partial efficacy against CNS neuropathology [34]. Nevertheless, we cannot exclude the possibility that even this relatively low efficacy within the CNS following neonatal IV gene therapy has contributed in part to the rescue of neuromuscular phenotypes we have documented in this study.

Although systemic gene therapy appears capable of treating pathology in the neuromuscular system (this study) and within the bowel [34] in CLN1 disease, this approach has its limitations. There are consistent reports of hepatocellular carcinoma following intravenous delivery of AAV9 [23,51,52], and new vectors and capsid variants that do not transduce the liver may be required. In addition, despite reports that AAV9 may transduce the CNS following neonatal delivery or even weeks later [53,54], our data suggest that the effects of intravenous gene therapy upon the brain are relatively limited, especially if delivered after the blood–brain barrier has fully formed [34]. Furthermore, CNS-directed therapies, such as intrathecal or intracerebroventricular enzyme replacement or gene therapy delivered via the same routes, may be insufficient to fully address the multisystemic nature of the disease in peripheral tissues. Based on our experience of treating disease both within and beyond the CNS [18,23,34,43,44], it is likely that dual delivery of gene therapy to treat the brain and body simultaneously will be needed to maximize efficacy. The systemic dose used in this study provides proof-of-concept for an approach designed to achieve robust peripheral transduction in this rapidly progressing mouse model. Translation of such strategies to the clinic will likely require optimization to enable therapeutic efficacy at lower vector doses, potentially through capsid engineering, refined tissue targeting, modified enzyme delivery, or the use of combined central and peripheral delivery routes.

Our findings have several broader implications. First, they reinforce the need to evaluate peripheral tissues in preclinical and clinical studies of NCLs and other neurodegenerative lysosomal diseases. Second, they highlight NMJ degeneration and skeletal muscle atrophy as potential contributors to late-stage motor decline in CLN1 patients, particularly in cases where CNS disease is partially stabilized. Third, they suggest that combination strategies targeting both central and peripheral compartments, such as dual-route gene therapy, may be necessary to optimize therapeutic efficacy and quality of life.

In conclusion, our study expands the known pathological spectrum of CLN1 disease to include the neuromuscular junction and skeletal muscle. These findings emphasize the systemic nature of this lysosomal disorder and the importance of targeting peripheral tissues in therapeutic design. Continued investigation of the timing, mechanisms, and functional consequences of these peripheral changes will be essential to developing holistic, effective treatments for CLN1 disease.

### Study Limitations and Future Directions

While our findings provide new insights into peripheral nerve and neuromuscular pathology in murine CLN1 disease, several limitations should be acknowledged. First, our analyses were limited to the symptomatic disease stage onwards, and further studies are needed to define the onset of these novel pathologies in relation to events in the CNS. Second, we suggest a more detailed investigation of the impact of disease upon synaptic organization, the true extent of denervation of the NMJ, and whether more subtle pathological effects upon peripheral nerves are apparent. Third, although we observed significant histological changes, we did not perform functional assessments of motor strength or muscle contractility, which will be important to establish the physiological relevance of these findings and whether any such defects are treatable. Fourth, the mechanisms driving peripheral pathology remain unclear, and whether these changes are due to cell-autonomous effects of PPT1 deficiency at the NMJ upon skeletal muscle itself or secondary to CNS dysfunction warrants further investigation.

Future studies should explore whether similar NMJ and muscle abnormalities occur in additional forms of NCL and other lysosomal storage disorders. It will also be important to determine the impact of timing, route, and dosage of gene therapy on peripheral outcomes, and whether later intervention can still preserve or restore neuromuscular function, alongside strategies to improve the efficacy of gene therapy within the neuromuscular system. Ultimately, combining central and systemic treatment approaches may prove essential for achieving full therapeutic benefit in CLN1 disease.

## 4. Materials and Methods

### 4.1. Mice

*Ppt1^−/−^* mice [55] and wild-type (WT) mice were kept separately on a congenic C57Bl/6J genetic background at Washington University School of Medicine. The mice were given a standard rodent diet (Purina Rodent Diet 5053, Richmond, IN, USA) and had access to water ad libitum under a 12 h light/dark cycle. The numbers analyzed are specified in the figures; all studies utilized an equal distribution of males and females. These investigations adhered to ARRIVE guidelines and were conducted under protocols 2018-0215, 21-0292, and 24-0232, which were approved by the Institutional Animal Care and Use Committee (IACUC) at Washington University School of Medicine, St. Louis, MO, USA.

### 4.2. Sciatic Nerve Staining and Analyses

Harvested nerve samples (*n* = 6 per group) were preserved in 3% Electron Microscopy grade glutaraldehyde (Polysciences, Warrington, PA, USA) at 4 °C, subsequently postfixed with 1% osmium tetroxide, and systematically dehydrated using ethanol. The specimens were then embedded in Araldite 502 (Polysciences) and sliced into 1 μm cross-sections with an ultramicrotome (LKB III Produkter A.B., Bromma, Sweden). For visualization via light microscopy, the sections were stained with 1% toluidine blue dye, a reliable technique for quantitative assessment of peripheral nerves, and mounted onto slides [56,57]. Quantitative analysis was conducted using a semi-automated digital image analysis system integrated with Clemex PE software, version 9.2, specifically tailored for nerve morphometry (Clemex Technologies Inc., Longueuil, QC, Canada) [56]. Clemex PE facilitates the creation of analytical methods through sequential selection of standardized workflows, thereby eliminating the necessity for programming. The workflow implemented is derived from our previous studies and can be obtained from us in Clemex PE upon request [35]. In summary, the sectioned nerve images were partitioned into non-overlapping frames of 100 × 100 μm^2^ and assessed using eight-bit plane digital pseudo-coloring along with thresholding-based algorithms to identify myelinated axons, as well as to calculate their density, area, myelin thickness, and diameters [56,57].

### 4.3. Immunofluorescence and Quantitative NMJ Analyses

Extensor digitorum longus (EDL) muscles from *Ppt1^−/−^* and control WT mice were carefully dissected and preserved overnight at 4 °C in fresh 4% paraformaldehyde for subsequent histological examination. EDL wholemounts and longitudinal frozen sections (25 µm thickness) of muscle were subjected to immunofluorescence staining as previously outlined [23,40], involving incubation with primary antibodies overnight at 4 °C and secondary antibodies for an hour at room temperature (Supplementary Table S1). Additionally, endplates were labeled with α-Bungarotoxin-Alexa 555 (α-BTX), which specifically binds to acetylcholine receptors (AChRs) present in the postsynaptic membrane [56]. Slides were prepared using Vectashield mounting medium containing DAPI for nuclear labeling. NMJs were captured *en face* using an AxioImager M2 fluorescent microscope (Carl Zeiss, Oberkochen, Germany) and analyzed with NIH ImageJ, Version 1.53K. For the analysis, over 100 NMJs per muscle were quantified from both EDL wholemounts and frozen sections, with at least 12 random sections stained per muscle [23,58]. Three components of the NMJ were examined: tSCs (using S100B antibody for all glial cells), presynaptic nerve terminals (using Neurofilament 200 antibody, NF200), and postsynaptic motor endplates (using α-BTX). To assess the percentage of normal versus atypical endplates, the number of fragments per endplate was recorded [23,58]. Endplates were classified as normal if AChRs, stained with α-BTX, formed a continuous “pretzel-like” architecture with ≤7 fragments and a consistent overall size. An NMJ was deemed fully innervated if nerve and endplate clusters were aligned over ≥75% of their length. An NMJ was considered partially innervated if the nerve terminal only partially (<75%) covered the endplate stained with α-BTX. Denervation was characterized by the absence of neurofilaments overlapping the α-BTX staining. The percentage of innervated NMJs was adjusted to reflect the total number of NMJs within the microscopic field of view [23,58]. Control sections (either lacking primary or secondary antibody) were included in every staining type to assess for nonspecific staining. For all histological and immunofluorescence analyses, representative images are shown, while quantitative analyses were performed on multiple sections and multiple neuromuscular junctions or muscle fibers per mouse, as detailed in the figure legends.

### 4.4. Muscle Histology and Analyses

Immediately following decapitation, the quadriceps femoris were collected and preserved overnight at 4 °C in freshly prepared 4% paraformaldehyde [23]. The preserved tissues were embedded in paraffin blocks and sectioned at a thickness of 10 µm using a sledge microtome. The sections underwent deparaffinization and were stained with hematoxylin and eosin following standard protocols [23]. A minimum of 300 muscle fibers per mouse were analyzed in the quadriceps. Furthermore, the quadriceps femoris was stained for ß-laminin (Abcam, Cambridge, UK, rabbit anti-laminin, 1:200) to evaluate the extracellular deposits between muscle fibers and for PPT1 (Atlas, Tokyo, Japan, anti-PPT1, 1:200) to reveal the distribution of AAV9-hCLN1 transduced myofibrils expressing PPT1, followed by an AlexaFluor goat anti-rabbit 488 (Thermofisher, Waltham, MA, USA, 1:400). Details of all antibodies are provided in Supplementary Table S1.

### 4.5. Intravenous Gene Therapy

Injections employed an AAV9 virus expressing human CLN1 (hCLN1), defined as AAV9-hCLN1. The AAV vector used in this study was a single-stranded AAV9 (ssAAV9) construct encoding the human PPT1 cDNA under the control of the CAG promoter. This vector was the same as that used in our previously published studies [18,28,29,30,31,32,43], containing the same expression cassette, promoter, and AAV9 capsid. An AAV9-GFP expressing vector in which the PPT1 cDNA sequence was replaced with GFP was used as a control for vector expression. Viruses were packaged at the University of North Carolina Vector Core. Virus injections 1.5 × 10^11^ vg/mouse were administered into the superficial temporal vein of neonatal (P1) mice that were under hypothermic anesthesia [23,34]. The neonatally injected mice were then returned to their mothers, nurtured until weaning, and kept either until the mice were symptomatic (5 months) or until the anticipated disease endstage (7 months), the time point at which most analyses were performed.

### 4.6. Statistical Analyses

Statistical analyses were performed using GraphPad Prism version 10.4.1 for MacOS (GraphPad Software, San Diego, CA, USA). A one-way ANOVA with a post hoc Bonferroni correction was used for comparison between three groups or more. Unpaired *t*-tests were used for comparisons between two groups. A *p*-value of ≤0.05 was considered significant.

## 5. Conclusions

Our study reveals that CLN1 disease, long regarded as a CNS-centered disorder, also displays profound pathology at the neuromuscular junction and within skeletal muscle. These peripheral defects, characterized by loss of terminal Schwann cells, apparent NMJ denervation, and muscle fiber atrophy, occur independently of overt peripheral nerve axonal degeneration or demyelination. These data represent a previously unrecognized aspect of disease pathogenesis, and we show that early systemic AAV9-hCLN1 gene therapy can mitigate many of these abnormalities, transducing a subset of myofibrils and preserving NMJ innervation and skeletal muscle structure. These findings broaden the known pathophysiology of CLN1 disease and underscore the need to expand therapeutic strategies beyond the CNS. Targeting both central and peripheral compartments will be essential to achieving meaningful and lasting benefits for individuals affected by this devastating disease.

## Data Availability

The original contributions presented in this study are included in the article/Supplementary Materials. Further inquiries can be directed to the corresponding author.

## Supplementary Materials

The following supporting information can be downloaded at: https://www.mdpi.com/article/10.3390/ijms27073080/s1.

## Abbreviations

CNS	central nervous system
tSC	terminal Schwann cell
NMJ	neuromuscular junction
NCLs	neuronal ceroid lipofuscinoses
PNS	peripheral nervous system
AAV	adeno-associated viral vector
AAV9	adeno-associated viral vector, serotype 9
WT	wild type
IACUC	Institutional Animal Care and Use Committee
EDL	Extensor digitorum longus
AChRs	acetylcholine receptors
α-BTX	alphabungarotoxin
