# Supplementary material for: Systemic AAV9 Gene Therapy Mitigates Neuromuscular Junction Degeneration and Muscle Atrophy in a Mouse Model of CLN1 Disease
Source: Int J Mol Sci. 2026 Mar 28;27(7):3080. doi: 10.3390/ijms27073080 (PMC13073675; doi:10.3390/ijms27073080)
Supplement: Supplementary file 1 [file ijms-27-03080-s001.zip › Table S1.pdf]

Supplementary Table S1 Antibodies used

Antibody	Concentration	Catalog number	Source
Rabbit anti-S100 beta	1:300 (mouse NMJ)	S2655	Sigma<br>RRID: AB_10641800
Rabbit anti- NF 200	1:400 (mouse NMJ)	N4142	Sigma Millipore<br>RRID: AB_477272
Rabbit anti-Laminin	1:200 (mouse muscle)	ab11575	Abcam<br>RRID: AB_298179
Rabbit anti PPT1	1:200 (mouse muscle)	HPA021546	Atlas<br>RRID: AB_1855667
Alpha-Bungarotoxin Alexa Fluor 555 conjugate	1:1000 (mouse NMJ)	B35451	ThermoFisher Scientific<br>RRID: AB_2617152
Alexa Fluor goat anti-rabbit 488	1:1000 (mouse NMJ & muscle)	A-11008	ThermoFisher Scientific (Invitrogen)<br>RRID: AB_143165
